# Relationship Between Maternal Serum Bile Acid Levels and Fetal Cardiac Troponin-I Levels in Asymptomatic Pregnant Patients at Term: A Cross-sectional Observational Study

**DOI:** 10.7759/cureus.5508

**Published:** 2019-08-28

**Authors:** Julie R Whittington, Lee R Allen, Christopher S Ennen, Craig M Zelig

**Affiliations:** 1 Obstetrics and Gynecology, University of Arkansas for Medical Sciences, Little Rock, USA; 2 Obstetrics and Gynecology, Naval Medical Center, Portsmouth, USA; 3 Obstetrics and Gynecology, Albany Medical Center, Albany, USA

**Keywords:** intrahepatic cholestasis of pregnancy, pruritis, bile acids, asympatomatic hypercholanemia

## Abstract

Objective

The objective of our study was to determine if a correlation exists between maternal total bile acid levels, degree of maternal pruritus, and fetal cardiac troponin-I levels in asymptomatic patients without a diagnosis of intrahepatic cholestasis of pregnancy.

Study design

In this cross-sectional observational study, patients were enrolled at the time of the scheduled term cesarean section. Maternal blood was drawn for fasting total bile acid levels and cord blood was collected for fetal cardiac troponin-I levels. Pruritus during pregnancy was quantified by the patient on a visual analog scale (VAS). Correlation coefficients between these variables were calculated.

Results

There was not a positive correlation between any of the primary variables studied (pruritis, total bile acid, cardiac troponin I). Pearson's R between total bile acid and cardiac troponin I was -0.058 (weak correlation in the opposite direction), and between total bile acid and pruritus severity, it was 0.031.

Conclusion

In patients without intrahepatic cholestasis of pregnancy, higher levels of maternal total bile acids did not correlate with increased cardiac troponin-I (fetal cardiomyocyte damage) or increased pruritus. This supports the current theory that the adverse outcomes associated with intrahepatic cholestasis of pregnancy require a threshold value of total bile acids, one high enough to cause clinically significant maternal pruritis.

## Introduction

Intrahepatic cholestasis of pregnancy (ICP) is a relatively rare condition (1-5/1000) that is associated with adverse pregnancy outcomes, such as preterm delivery, meconium staining of amniotic fluid, and fetal asphyxial events, including intrauterine fetal demise (IUFD). The occurrence of IUFD in ICP pregnancies is often not predicted by antepartum fetal testing and typically occurs late in the third trimester in 2% to 3% percent of affected pregnancies. The only symptom of ICP is intractable pruritus. ICP is diagnosed in pregnancy when severe pruritus is accompanied by an elevated fasting serum total bile acid (TBA) level, typically greater than 10 micromoles per liter [1-8].

The leading theory proposed for the increased IUFD rate in ICP is that the elevated level of bile acids in the fetal compartment causes fetal cardiac damage. Studies have shown fetal arrhythmias in pregnancies complicated by ICP and in vitro studies have demonstrated bile acid-induced arrhythmias in fetal cardiomyocytes [9-11]. Fetal cardiac damage can be detected through measurement of various cardiac enzymes, including troponin-I [12-15]. A recent large prospective study demonstrated elevated levels of cardiac troponin-I (cTnI) in the cord blood at the time of delivery in pregnancies affected by ICP [16]. Cardiac damage in these neonates was confirmed with fetal echocardiography using the Tei index, which was described in 2003 [17]. This clinical study provided support for the in vitro studies of cardiomyocyte damage as a mechanism of morbidity in pregnancies affected by ICP.

While pruritus is required for the diagnosis of ICP, there is no correlation between the degree of pruritus and the severity of the disease. In fact, up to 50% of pregnant woman suffer from various degrees of pruritus but only those with a concomitant elevation of fasting total bile acids are diagnosed with ICP. Once the diagnosis of ICP is made, specific treatment algorithms are followed to reduce maternal symptoms and minimize the risk of stillbirth. Interventions include use of ursodeoxycholic acid, antepartum fetal testing, and induction of labor at 36-38 weeks gestational age, depending on the severity of the bile acid elevation [18-21]. The risk of stillbirth is more common with a bile acid level in excess of 40 micromoles per liter and these patients are often monitored more closely [22].

Not all patients with bile acid levels associated with stillbirth and other pregnancy complications have significant pruritus and hence are never screened. A prospective study of 411 healthy asymptomatic pregnant patients detected a sub-population of patients with bile acid levels two-fold higher than the mean. This subgroup comprised 9% of the study population. Importantly, these hypercholanemic patients (as they were dubbed by the authors of the study) had proportions of molecular bile acid subtypes similar to those seen in ICP patients and not those seen in normal pregnant patients [23]. The objective of our study was to determine if there exists a population of patients without significant pruritus but with bile acid elevations significant enough to affect fetal cardiac cells, as defined by an elevation in cord blood cardiac troponin-I.

## Materials and methods

Inclusion criteria and study procedures

From August 2014 to June 2016, patients were recruited at Naval Medical Center Portsmouth at the time of the scheduled cesarean delivery. Inclusion criteria included women presenting for scheduled cesarean delivery, greater than or equal to 37 weeks gestational age, who were eligible for care at our facility. Exclusion criteria included active labor, current or prior diagnosis of intrahepatic cholestasis of pregnancy, or known major fetal anomalies. Patients were considered to have intrahepatic cholestasis of pregnancy when the patient endorsed itching and had laboratory confirmation of the total bile acid level exceeding 10 micromoles per liter.

The patient’s fasting TBA level was drawn on admission, prior to surgery. Total bile acids (glyco and taurochenodeoxycholic acid) were analyzed at Labcorps (Burlington, NC, US). At the time of delivery, umbilical cord blood was collected for cTnI measurement and sent to our institution’s lab. cTnI levels were measured in micrograms per milliliter. Demographic data collected included age, parity, and race as well as co-existing diseases (e.g. hypertension, diabetes, gallbladder disease, etc.). Outcomes of delivery (e.g. weight, gestational age, and delivery complications such as meconium, Apgar (Appearance, Pulse, Grimace, Activity, and Respiration) scores, cord pH, and Neonatal Intensive Care Unit (NICU) admission) were also recorded. Patients were also asked whether or not they experienced pruritus during their current pregnancy, how severe it was (on a visual analog scale), how often it occurred (how many days per week), and whether or not they took any medication for it. The visual analog scale was originally used for an estimation of pain, where patient marked their symptoms on a horizontal line where the left side of the line is no pain and the right line is the worst pain imaginable. The visual analog scale for pruritis severity is easy and reliable to use to assess itching severity [24].

Statistical analysis

A power analysis was performed to determine the needed sample size. The prospective study by Zhang et al. demonstrated elevated troponin-I levels in the cord blood of pregnancies affected by ICP as compared to unaffected pregnancies. The levels were 0.92±0.23 micrograms/liter in the ICP group versus 0.52±0.10 micrograms/liter in the normal group [16]. We assumed conservatively that the effect we would observe in our asymptomatic patients would be half as great as those observed in the prior study, therefore, we wanted a sample size to detect an average troponin-I level of 0.72. Based on a prior study, we expected that 9% of our patients would have asymptomatic elevations in their bile acid levels [23]. This equated to a ratio of 10.1:1 (unaffected to affected). In a smaller sample of asymptomatic patients without ICP, 40% of patients had asymptomatic hypercholanemia with bile acids greater than 11 micromoles per liter [25].

To achieve a power of 80% with a 95% two-sided confidence interval (alpha=.05) to detect the difference in troponin-I levels defined above requires a sample size of n=123 per Openepi Version 3 (student t-test). We recruited 170 patients to account for errors in our estimate and patient dropout.

The primary result was the difference in cTnI levels in the cord blood of patients with normal maternal TBA levels versus those with elevated maternal TBA levels (unpaired student t-test). Elevated maternal TBA level was defined as greater than 10 micromoles per liter. In addition, we calculated correlation coefficients between maternal TBA levels and fetal (umbilical cord) cTnI levels. Logistic regression was carried out to determine independent risk factors for elevated cTnI levels (indicating cardiomyocyte damage). 

This cross-sectional observational study protocol was approved by the Naval Medical Center Portsmouth Institutional Review Board in compliance with all applicable Federal regulations governing the protection of human subjects.

## Results

We enrolled 170 subjects; 7 patients enrolled did not have either lab value obtained (total bile acids or troponin-I) and were, therefore, excluded, leaving the data for 163 subjects for analysis. Demographic data for the patients is shown in Table 1. 

**Table 1:** Maternal characteristics Data are n (%) or mean±SD unless otherwise specified.

Characteristic	Total Cohort (n=163)
Age (y)	29.6±4.5
Multiparous	145 (89)
Gestational age (wk)	39.1±0.6
Race	
White	94 (58)
Hispanic	19 (12)
Black	31 (19)
Asian	5 (3.1)
Native American	6 (3.6)
Pacific Islander	8 (4.9)
Medical complications	
Chronic hypertension	8 (4.9)
Gestational hypertension	2 (1.2)
Cholelithiasis	1 (0.61)
Preeclampsia	1 (0.61)
Anemia	12 (7.4)
Hypothyroidism	11 (6.7)
Pregestational diabetes	2 (1.2)
Gestational diabetes	19 (12)
Obesity	37 (23)
Anxiety / depression	31 (19)
Asthma	15 (9.2)
Migraines	11 (6.7)
Pruritis during pregnancy	
Pruritis present	80 (49)
Days per week	4.6 (2.4)
Medication taken for pruritis	13 (7.9)

Obstetric and neonatal characteristics are shown in Table 2. 

**Table 2:** Obstetric and neonatal characteristics Data are n (%) or mean±SD unless otherwise specified. BE = base excess, LOS = length of stay, NICU=neonatal intensive care unit *Cord gases available for 23 subjects.

Characteristic	Total Cohort (n=163)
Birth weight (grams)	3500±498
5-min Apgar score < 5	0 (0)
Estimated blood loss (milliliters)	707±152
Meconium stained amniotic fluid	1 (0.61)
Umbilical artery pH *	7.2±0.10
Umbilical artery BE (millimoles/liter)	-3.5±2.9
NICU admission	13 (7.9)
NICU LOS (days)	7.1±(5.2)
Perinatal death	0 (0)

**Figure 1:**
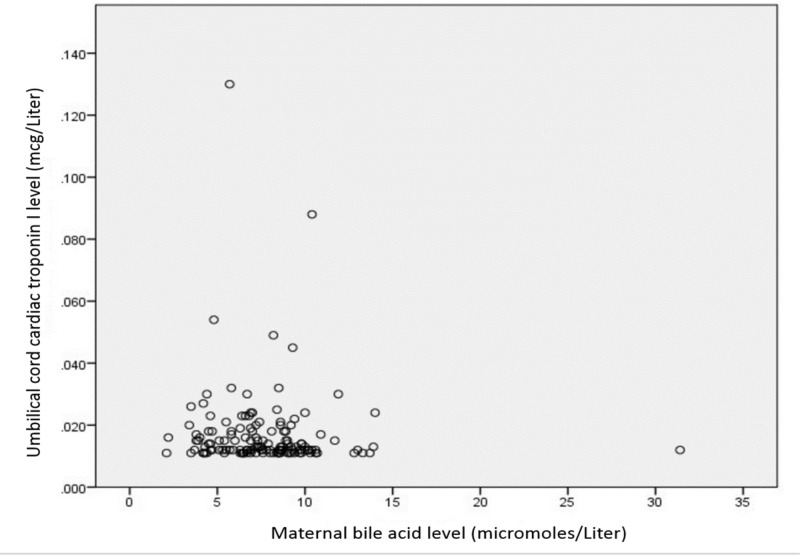
There was no correlation between these maternal total bile acid levels (TBA) and fetal cardiac troponin-I (cTnI) with a Pearson’s r coefficient of -.058.

There was not a positive correlation between any of the primary variables studied. Pearsons R between TBA and cTnI was -.058 (weak correlation in the opposite direction, see Figure 1).

**Figure 2:**
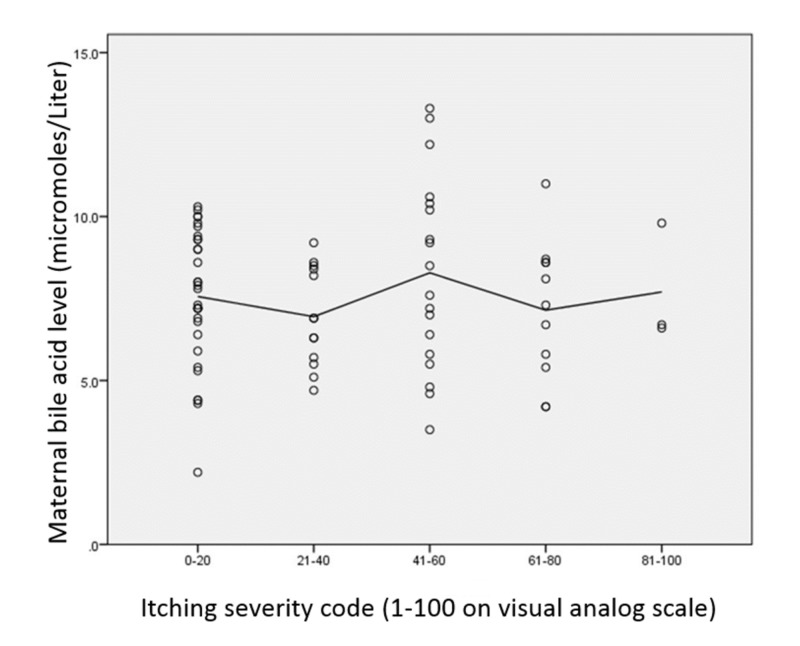
There was also no apparent correlation between the severity of itching on the visual analog scale and maternal total bile acid level (TBA) with a Pearson’s r coefficient of .031

Pearsons R between TBA and pruritus severity on the visual analog scale was .031 (see Figure 2). 

The mean troponin level for patients with bile acids less than 10 was .0169 ±.0126 micrograms per milliliter. The mean troponin for patients with bile acids 10 or greater was .0177±.0177 micrograms per milliliter.

**Figure 3:**
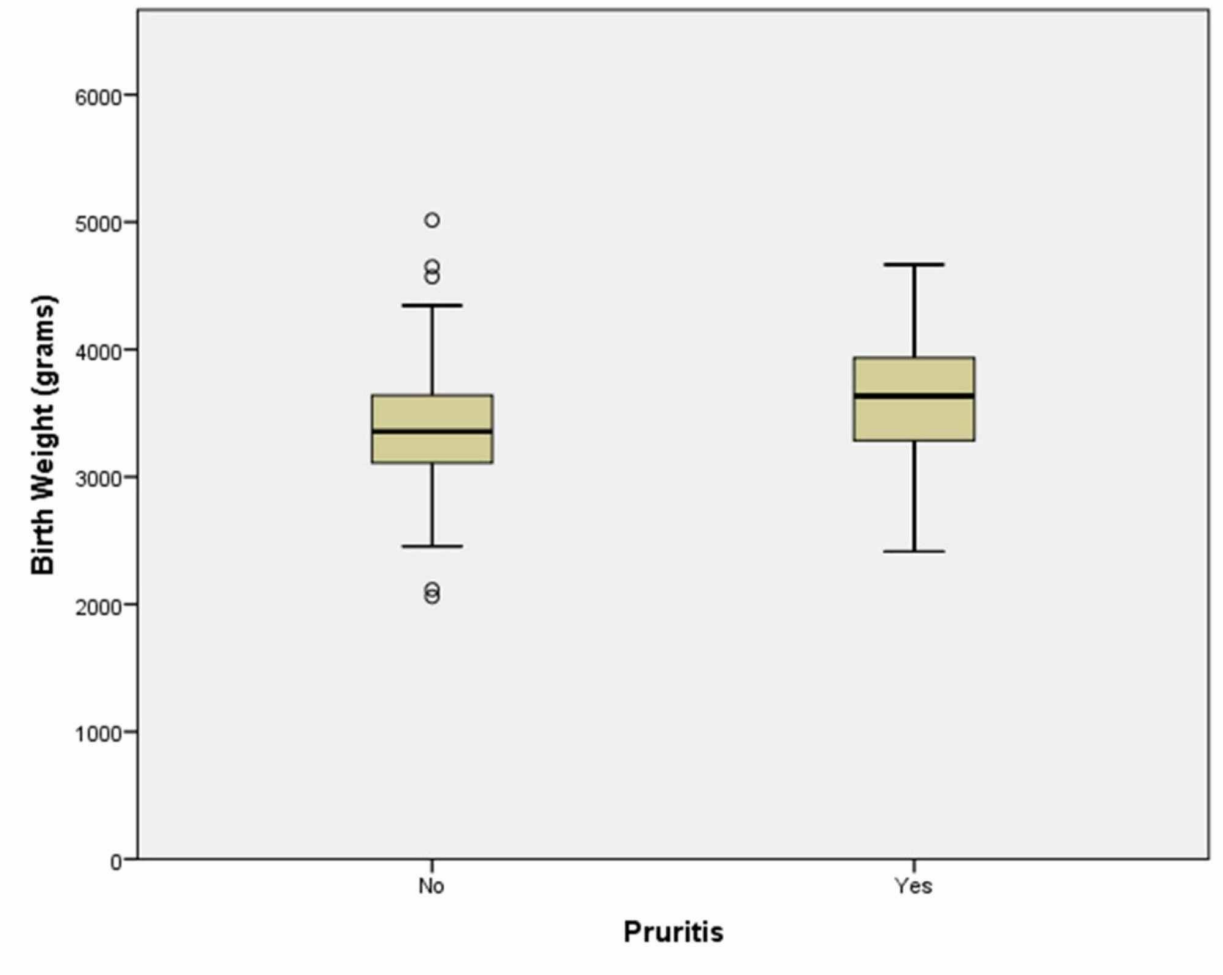
The only statistically significant difference we found is mothers with larger infants tended to have pruritis as opposed to mothers with smaller infants.

There were no patients in our study with bile acids > 40 micromoles/L, the cutoff for moderate disease in patients diagnosed with ICP. The maximum bile acid level measured in our study was 31 micromoles/L and that patient did not have pruritis, therefore, he would be considered as having asymptomatic hypercholanemia. Women with itching were noted to have larger infants with a p-value of 0.009 (Figure 3). 

## Discussion

We did not find a significant increase in fetal cardiac troponin-I in patients without a diagnosis of intrahepatic cholestasis of pregnancy. That is, there does not seem to be a population of patients without significant pruritus with bile acid elevations high enough to cause fetal cardiac strain. Even in patients with bile acids >10 micromoles/liter (hypercholanemic patients), there was not a significant increase in fetal cardiac troponin-I. We did find more asymptomatic hypercholanemic patients than anticipated. Based on a prior study, 9% of pregnant patients are hypercholanemic, defined as bile acids greater than 6 micromoles per liter, however, in our study population, 19.6% of patients had elevated total bile acids with levels >10 micromoles/L [23]. This is less than the 40% of patients observed in a previous study with asymptomatic hypercholanemia of pregnancy [25]. No patients in our study had a TBA level greater than 40 micromoles/L.

Intrahepatic cholestasis of pregnancy is a serious condition associated with multiple complications in pregnancy. Management of intrahepatic cholestasis of pregnancy often involves early delivery, thus increasing the risks of prematurity over the less likely, but grave possibility of IUFD. In a recent retrospective study, the level of bile acids correlated with adverse pregnancy outcomes; severe intrahepatic ICP (>100 micromoles/L) conferred an increased risk of spontaneous preterm birth, perinatal death, and meconium-stained amniotic fluid [26]. Thus far, no test has proven useful in predicting fetal death in this condition. It is interesting that many subjects in our study had mildly elevated total bile acids without antenatal complications. It may be reasonable that those with mild intrahepatic cholestasis of pregnancy may be delivered later in pregnancy than those with moderate to severe intrahepatic cholestasis of pregnancy. However, as risk does increase with duration of pregnancy, this does not extend far. One possible consideration could be to deliver those with TBAs > 100 as early as 36 0/7, for those with levels between 40 and 100 at 37 0/7 through 37 6/7, and for those under 40 between 37 0/7 and 38 6/7 weeks gestation. Another potential protocol could be to deliver all those with TBAs > 40 at 36 0/7 through 37 6/7 and those with less than 40 at 37 0/7 through 37 6/7. Others suggest that delivery of all patients with ICP at 36 weeks gestation would be beneficial [27]. They do not specifically address a stratified approach by TBA level. This is an area where further research could provide more guidance about the details of delivery timing.

The strengths of our study include the number of subjects and that all patients were fasting given their nil per os status in preparation for surgery. The Strengthening the Reporting of Observational Studies in Epidemiology (STROBE) guidelines were also followed. The limitations of our study include that there are no patients with bile acid elevations greater than 40, which has been a cut-off for moderate to severe disease. Additionally, we were looking at patients without a diagnosis of ICP but specifically measuring their TBA levels and ascertaining their amount of pruritus and found no correlation of those with a marker for fetal cardiomyocyte damage. One prior study did demonstrate an elevated level of cTnI in the cord blood of neonates at the time of delivery from mothers with ICP. Our study suggests that either there is a threshold that must be met for a patient to become sufficiently symptomatic to thus warrant a diagnosis of ICP in order for cTnI to be elevated or that the two may still be unrelated. Further work could look again at the correlation in patients with ICP to clarify that point. Whether the current hypothesis that ICP leads to potentially fatal arrhythmias in the fetus remains inconclusive.

## Conclusions

Based on our findings, elevated bile acids in the absence of clinical ICP (asymptomatic hypercholanemia of pregnancy) is not associated with significant elevations in fetal troponin levels. Therefore, pregnant women with asymptomatic bile acid elevation may not have the same risks for adverse pregnancy outcomes as patients with a diagnosis of ICP. There is no reason to check bile acid levels in pregnant women without symptoms.
